# Quantitative Peptidomics of Mouse Brain After Infection With Cyst-Forming *Toxoplasma gondii*

**DOI:** 10.3389/fimmu.2021.681242

**Published:** 2021-07-22

**Authors:** Chun-Xue Zhou, Min Gao, Bing Han, Hua Cong, Xing-Quan Zhu, Huai-Yu Zhou

**Affiliations:** ^1^ Department of Pathogen Biology, School of Basic Medical Sciences, Cheeloo College of Medicine, Shandong University, Jinan, China; ^2^ State Key Laboratory of Veterinary Etiological Biology, Key Laboratory of Veterinary Parasitology of Gansu Province, Lanzhou Veterinary Research Institute, Chinese Academy of Agricultural Sciences, Lanzhou, China; ^3^ College of Veterinary Medicine, Shanxi Agricultural University, Taigu, China

**Keywords:** *Toxoplasma gondii*, mouse, brain, peptidomics, neuropeptide

## Abstract

*Toxoplasma gondii* is an obligate intracellular parasite capable of establishing persistent infection within the host brain and inducing severe neuropathology. Peptides are important native molecules responsible for a wide range of biological functions within the central nervous system. However, peptidome profiling in host brain during *T. gondii* infection has never been investigated. Using a label-free peptidomics approach (LC–MS/MS), we identified a total of 2,735 endogenous peptides from acutely infected, chronically infected and control brain samples following *T. gondii* infection. Quantitative analysis revealed 478 and 344 significantly differentially expressed peptides (DEPs) in the acute and chronic infection stages, respectively. Functional analysis of DEPs by Gene Ontology suggested these DEPs mainly originated from cell part and took part in cellular process. We also identified three novel neuropeptides derived from the precursor protein cholecystokinin. These results demonstrated the usefulness of quantitative peptidomics in determining bioactive peptides and elucidating their functions in the regulation of behavior modification during *T. gondii* infection.

## Introduction

Human infection with the obligate intracellular parasite *Toxoplasma gondii* has been considered an underestimated threat (1, 2). People become infected with *T. gondii* by eating undercooked contaminated meat or ingestion of oocysts shed by infected cats (3). Although it is estimated that approximately 30% of the world’s human population carry this parasite, most people show no obvious clinical symptoms as the immune system can suppress parasite replication (4). However, *Toxoplasma* can be reactive in immunocompromised individuals, such as AIDS patients and transplant recipients, leading to retinochoroiditis, encephalitis and even death (5). Worthy to mention, unborn children might undergo diseases of the nervous system and eyes if their mothers are newly infected with *Toxoplasma* during or just before pregnancy (6). Unfortunately, there are no commercial vaccines that can stop or significantly lessen the chances of *Toxoplasma* infection.

Usually, an infected host especially rodents will undergo two phases, including acute infection and chronic infection. Acute infection is characterized by rapidly replicating tachyzoites which can infect all nucleated cells and disseminate throughout the body *via* bloodstream (7). The protozoan parasite will then differentiate into slow-growing bradyzoites that will persist in the host muscle and brain for the entire life (8). While the combination of sulfadiazine and pyrimethamine is highly effective against the tachyzoites, neither it, nor any of the other currently used drugs is capable of eradicating the cyst form of *T. gondii*. In humans, *T. gondii* infection has been linked to several mental diseases, such as schizophrenia, Parkinson’s disease (PD) and bipolar disorders (9–11). There have been numerous studies that have shown that *T. gondii *infection is associated with specific behavioral modifications in animals and humans. For example, infected rats and mice exhibited a significant decreased aversion to cat urine, and they even showed an unexpectedly attraction (12). Some previous studies found *T. gondii* even has an effect on suicide attempts and road rage (13, 14). Johnson et al. demonstrated that *T. gondii* infection is a positive predictive factor for entrepreneurial activity (15). Although it is known that behavioral changes are directly related to cyst burden, Jenniffer et al. revealed that neuroinflammation caused by chronic infection induced behavior changes regardless of the cyst burden (16). More evidence showed that *T. gondii* infection caused multiple neuronal changes, such as alteration of neurotransmitter and hormone levels (17, 18). However, the mechanisms underlying behavioral manipulation by* Toxoplasma* remain largely unclear.

Neuropeptides and peptide hormones are small biological molecular highly enriched in the central nervous system (CNS) and function as important regulators in neuronal connections. Growing evidences in animal models and humans have demonstrated the involvement of many neuropeptides in many physiological and psychological processes, including sleep, feeding and mood (19–21). Several studies have indicated that peptide variations are involved in neuropathological conditions, such as Alzheimer’s disease (AD) (22). Given the importance of the regulatory roles of neuropeptides and previous findings in behavior modifications induced by *T. gondii* infection, peptidome profiles, especially neuropeptide variations during *Toxoplasma* infection are very intriguing and worth exploring.

In the present study, we used a mass spectrometry (MS)-based peptidomics approach, in light of its reliability and sensitivity, to characterize a dynamic landscape wherein the neuropeptidome was affected by *T. gondii* infection. We found that remodeling of the brain peptidome by *T. gondii* infection was highly correlated with the severity of toxoplasmosis. These peptidomics analyses have the potential to provide an unbiased profiling and a deeper understanding of the underlying mechanisms of host neuropathology during toxoplasmosis.

## Materials and Methods

### Ethical Approval

All experimental procedures were conducted in accordance with the Animal Ethics Regulations and Procedures of the People’s Republic of China. This study was approved by the Animal Ethics Committee of the Lanzhou Veterinary Research Institute of the Chinese Academy of Agricultural Sciences (Permit No. LVRIAEC2016-06). All efforts have been made to alleviate suffering and reduce the number of animals used in the study.

### Animals and Parasites

Female BALB/c mice (6–8 weeks old) were purchased from the Lanzhou University, China. Female Kunming mice used for parasite maintenance were obtained from the Laboratory Animal Center of Lanzhou Veterinary Research Institute, Chinese Academy of Agricultural Sciences. All mice had free access to food and water throughout, and kept in specialized equipment under a 12-h light/dark cycle.

The low-virulence *T. gondii* type II Prugniaud (Pru) strain was maintained in our laboratory *via* oral inoculation of parasite cysts in Kunming mice. Following anesthetizing animals, brains of the infected mice at 40 days post infection were removed and homogenized in a tissue homogenizer under sterile conditions. The number of tissue cysts was counted and diluted to 100 cysts/ml in phosphate buffered saline solution (PBS) until further use.

### Animal Infections and Sample Collections

Female BALB/c mice were divided randomly into three groups (six in each group). Two groups were orally infected with ~10 freshly prepared tissue cysts *via* oral gavage. Six mock-infected (control) mice received the same amount of saline. All mice were monitored daily for clinical features throughout the course of infection. After anesthesia with 5% isoflurane gas, mice were rapidly decapitated, and brains from infected mice at 11 days [acute infection stage (AI)] and 35 days [chronic infection stage (CI)] post infection and control mice (Con) were removed as described previously (23). Brain tissues were immediately frozen in liquid nitrogen and pulverized to fine powder, which was then stored at −80°C until further use.

### Histopathological Analysis

Brain samples were fixed in 10% neutral buffered formalin solution and then were dehydrated by gradually soaking in alcohol and xylene and embedded in paraffin. The paraffin-embedded specimens were cut into 5-μm sections, stained with hematoxylin–eosin (H&E) and examined under a digital optical microscope (Olympus, Tokyo, Japan).

### Peptide Extraction

Brain tissues were manually homogenized in Dounce grinder containing cold lysis buffer (4% SDS, 15 mM Tris–HCl (pH 7.4), 10 mM sodium butyrate and protease inhibitor cocktail [Sigma-Aldrich, St. Louis, MO)]. The homogenized samples were centrifuged at 20,000*g* for 10 min at 4°C to remove the insoluble pellet. Supernatants were collected and diluted with 8 M urea to a final concentration less than 10 mg/ml. Dithiothreitol (DTT) with the final concentration of 10 mM was added, and the mixture were immersed into a water bath (56°C) for 1 h. To further remove larger proteins, the mixtures were applied to a 10 kDa molecular mass cutoff filter (Millipore, MA) and centrifuged at 14,000*g* for 15 min at 4°C. The filters were desalted in a Strata X C18 SPE column (Phenomenex) according to the manufacturer’s protocol. Peptides were eluted with acetonitrile (ACN)/water/formic acid (FA) solution (50:49.9:0.1; vol/vol/vol) and dried by vacuum centrifugation. Crude peptide samples were further dissolved in buffer A (5% ACN, 0.1%FA) for LC–MS/MS analysis.

### Mass Spectrometric Analysis

LC–MS/MS analysis was carried out as previously described with minor modifications (24). For peptidomics analysis, peptides were analyzed on a Q-Exactive spectrometer (Thermo Fisher Scientific, San Jose, CA) interfaced with a LC-20AD HPLC system (Shimadzu, Japan). Peptide samples were loaded onto a Trap column at 8 μl/min and separated by a C18 analytical column at 300 nl/min under gradient elution: 5% buffer B (%95 ACN, 0.1% FA) for 5 min, from 5 to 35% buffer B over 35 min, from 35 to 60% buffer B over 5 min, from 60 to 80% buffer B over 2 min, from 80 to 5% buffer B over 1 min, and 5% buffer B for 10 min.

Isolated peptide components from HPLC were subjected to nanoelectrospray ionization (NSI) followed by an orbitrap tandem mass spectrometer with data-dependent acquisition model detection. MS and MS/MS data were acquired automatically following an MS survey scan over m/z 350–1,800 m/z at a resolution of 70,000 for full scan and HCD (high-energy collisional dissociation) fragmentation at resolution of 17,500 for MS/MS measurements.

The peak lists from the MS/MS data were generated and applied to Maxquant 1.5.2.8 against the UniProt *Mus musculus* database (*Mus musculus*, 2016_06, 77,105 entries) (25). Precursor tolerances were set to 20 ppm with no enzyme specification. The peptides were searched with oxidation of methionine and acetylation on protein N terminus set as variable modification, and carbamidomethylation of cysteine set as a fixed modification. All data were searched as a single batch with PSM and protein FDR set to 1% using revert decoy mode. The match between runs and label-free quantification (LFQ) options were selected. Quantification of peptidomics data was performed using the MaxQuant LFQ algorithm.

### Bioinformatics Analysis

Gene Ontology (GO) analysis (http://www.geneontology.org) was performed for functional annotation and enrichment analysis (26). Pathway analysis was performed through the KEGG database (https://www.genome.jp/kegg/). Principal Component Analysis and Hierarchical Clustering were performed using online MetaboAnalyst 5.0 software (https://www.metaboanalyst.ca/) (27). The Search Tool for the Retrieval of Interacting Genes/Proteins (STRING) database was used to build protein–protein interaction networks (28). Cytoscape tool was used to visualize the interaction networks (29). Prepro-peptides are retrieved from the NeuroPedia, which is a neuropeptide encyclopedia of peptide sequences and spectral libraries of identified MS/MS spectra of homolog neuropeptides from multiple species (30). Potential proteases for neuropeptide production were predicted by online Proteasix tool (http://proteasix.org/) and the MEROPS database (https://www.ebi.ac.uk/merops/).

### Statistical Analysis

The Student’s *t* test was applied to calculate *p*-value to determine statistical difference between infection group and control group. Peptide features were considered to be significantly changed between two brain groups using *p-*value <0.05 and a fold change ≥1.5 or ≤0.67.

## Results

### Peptide Identification

**Figure 1:**
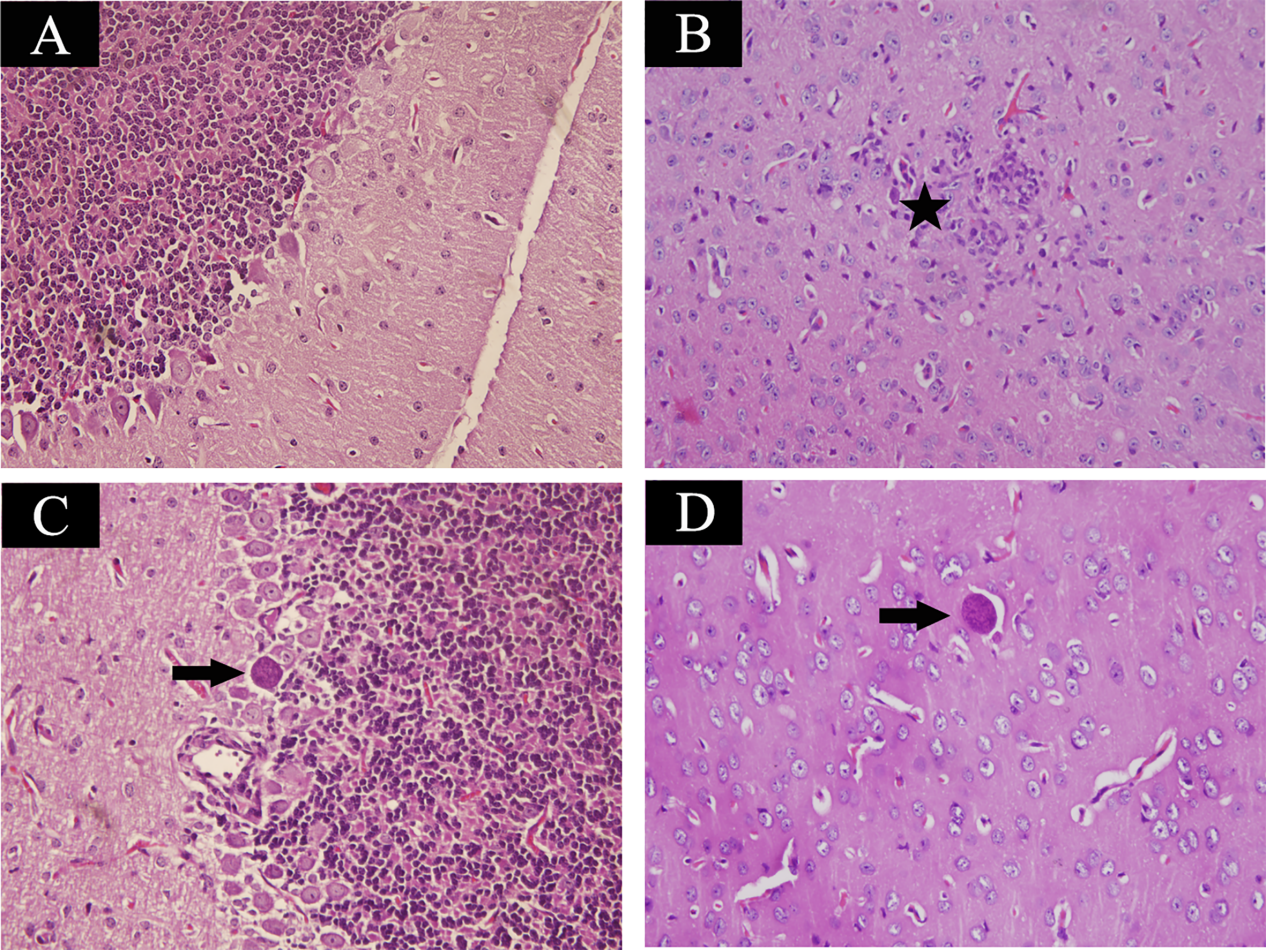
Histopathological lesions in mouse brain infected with *Toxoplasma gondii* Pru strain (10 cysts per mouse by oral route, H&E stain). **(A)** Brain histology of control mice without any pathological changes. **(B)** A section of brain from acutely infected mice. Pentagram indicates inflammatory cell infiltration. **(C, D)** Brain histology of infected mice at chronic infection phase. Arrow indicates the tissue cyst. Magnification (400×).

Mice at acute infection stage showed fever, anorexia, body weight loss, edema and messy hair. However, mice at 35 days post infection significantly increased their food intake and daily physical activity. Using H&E staining, brain samples were examined for histopathological damages caused by *T. gondii* infection. Control mouse brain did not show any histopathological abnormalities (**Figure 1A**). As shown in **Figure 1B**, infected mice at acute infection stage showed lymphocytic mononuclear inflammatory infiltrate. Tissue cysts filled with bradyzoites were found in mouse brain at 35 days post infection (**Figures 1C, D**).

**Figure 2:**
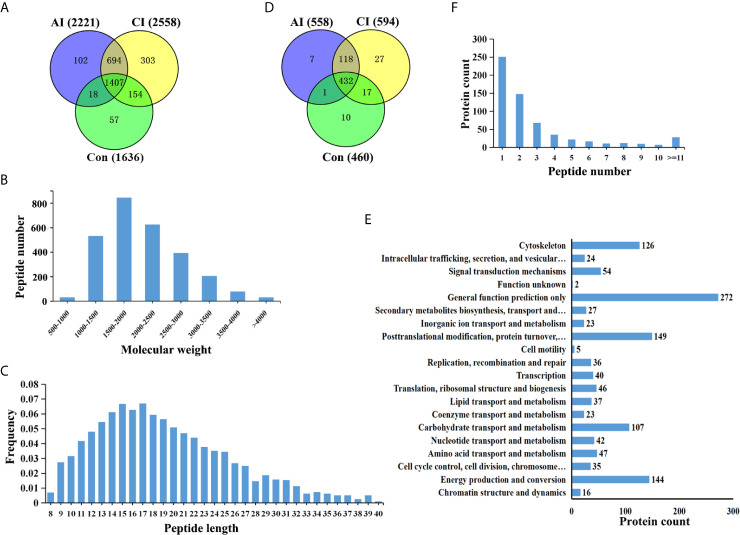
Identification of unique endogenous peptides in mouse brains. **(A)** Venn diagram of identified peptides in mouse brains. **(B)** Molecular weight distribution of identified peptides. **(C)** Frequency of peptide length distribution of identified peptides. **(D)** Venn diagram of identified precursor proteins. **(E)** Distribution of the number of unique peptides identified per protein. **(F)** COG analysis of precursor proteins.

**Figure 3:**
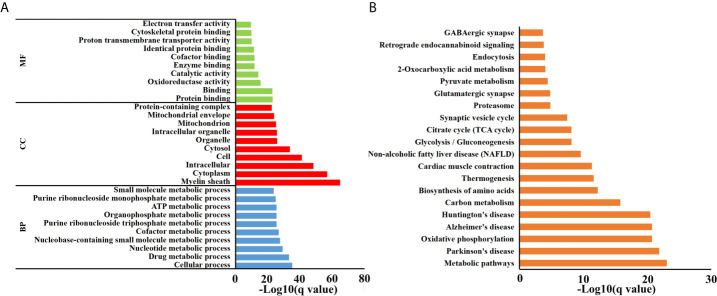
Functional analyses of precursor proteins. **(A)** Gene ontology (GO) analysis. Enriched terms are grouped by GO category biological process (BP), molecular function (MF) and cell component (CC). **(B)** The top 20 significantly enriched KEGG pathways.

Brain peptidome was analyzed for mice with *T. gondii* infections, which resulted in the identification of 2,735 unique peptides derived from 612 precursor proteins. As shown in **Figures 2A, D**, 2,221 fragments related to 558 proteins were found in the brain samples from the AI group, 2,558 peptide fragments of 594 proteins in the CI samples, and 1,636 fragments of 460 proteins in the Control group. Notably, 1,407 peptides and 432 precursor proteins were shared among the three groups of the samples. Compared to our previous study on serum peptidome (24), there were 62 peptides identified in both mouse brain and serum following *T. gondii* infection (**Figure S1** and **Table S1**). The molecular weight of identified peptides varied from 0.8 to 4.5 kDa and peaked at 1.5–2.0 kDa (**Figure 2B**). In the peptide length distribution, the maximum frequency was approximately 15 to 17, and then the peptide length increased as the frequency gradually decreased (**Figure 2C**). Precursor proteins were then subjected to COG analysis, and “general function prediction”, “post-translational modification and protein conversion” and “energy production and transformation” were the three most prominent COG categories (**Figure 2E**). As shown in **Figure 2F**, the majority of these identified precursor proteins contained less than three unique peptides. Gene Ontology (GO) analysis of these precursor proteins showed that ‘binding’, ‘oxidoreductase activity’ and ‘catalytic activity’ were significantly enriched (**Figure 3A**). Through KEGG analysis, quite a large number of precursor proteins were involved in progressive neurodegenerative disorders such as PD, AD, and Huntington’s disease (HD) (**Figure 3B**).

**Figure 4:**
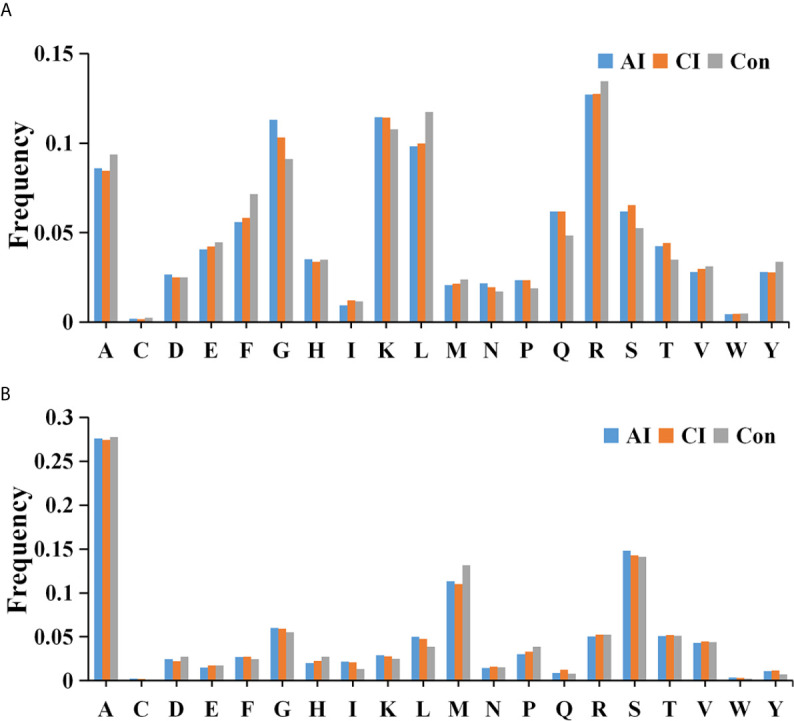
Frequency of the cleavage sites in the identified peptides. Cleavage specificity analysis of N-terminal peptide residue **(A)** or C-terminal peptide residue **(B)**.

We then searched for cleavage sites in all identified peptides to investigate functional changes in brains during *T. gondii* infection. As shown in **Figure 4A**, Arginine (R), Lysine (K), Glycine (G) and Leucine (L) were the most common cleavage sites of the C-terminal amino acid of the identified peptides. Alanine (A) was the most common cleavage sites at the N-terminus (**Figure 4B**).

### Brain Peptide Profiles Following *T. gondii* Infection

**Figure 5:**
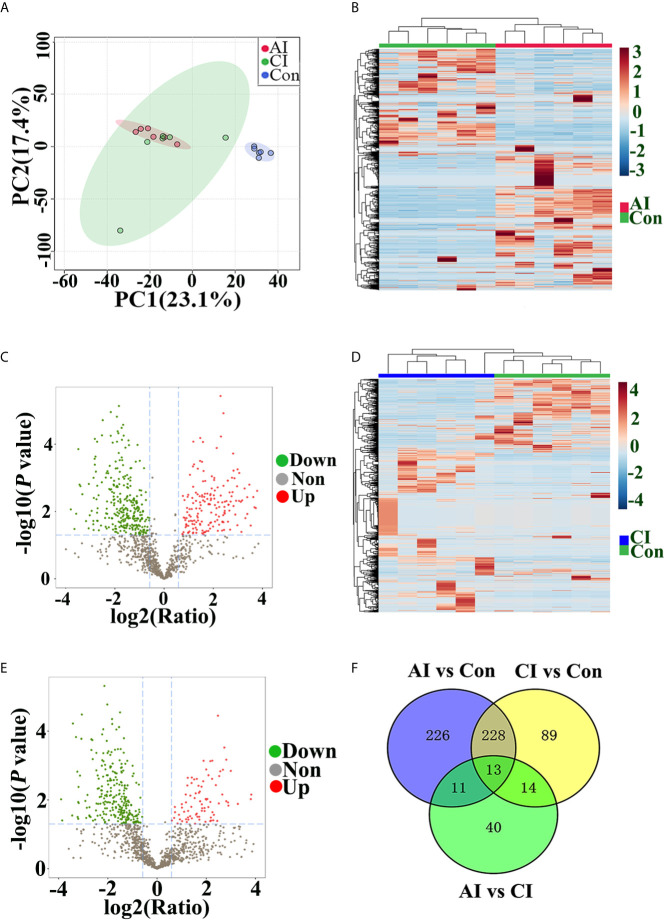
Peptide profile analysis among different groups. **(A)** PCA scores plot comparing the control group, acutely infected group and chronically infected group. **(B)** Heat map of peptides identified in both acutely infected group and control group. Columns were hierarchically clustered based on a complete linkage using Pearson correlation coefficients as the distance measure. **(C)** The volcano plot shows the individual statistically significant peptide between acutely infected group and control group. In this plot, the x-axis is log2 fold-change, which shows the direction of the change (negative scale is decrease and positive scale is increase) in the levels of peptide intensity, while the y-axis is the -log10 *p*-value, which shows the significance of the change. **(D)** Heatmap of peptides identified in both chronically infected group and control group. **(E)** The volcano plot shows the individual statistically significant peptide between chronically infected group and control group. **(F)** Venn diagram shows number of DEPs among different comparison pairs.

Multivariate data analysis of identified peptide was utilized to identify global differences during toxoplasmosis progression. As shown in **Figure 5A**, an unsupervised PCA scores plot for brain indicated an obvious separation along the first component (PC1), while samples from acutely infected group and chronically infected group intermixed. Heatmaps constructed based on normalized peptide intensity showed clear separation between acutely infected group and the control group (**Figure 5B**). Meanwhile, a total of 478 differentially expressed peptides (DEPs) were found in comparison between AI and Con, among which 184 were up-regulated and 294 were down-regulated (**Figure 5C**). As shown in **Figure 5D**, no obvious separation between chronically infected group and control group was found in hierarchical clustering. The volcano plot showed that levels of 88 peptides were increased, while expression levels of 256 peptides were decreased (**Figure 5E**). As shown in **Figure S2A**, hierarchical clustering did not clearly separate acutely infected samples from chronically infected ones. Compared to chronic infection group, 78 DEPs in the acute infection group were detected, among which levels of 69 proteins were upregulated, while nine proteins were downregulated (**Figure S2B**). As shown in **Figure 5F**, 13 endogenous peptides were shared among AI *vs.* Con, CI *vs.* Con and AI *vs.* CI. Detailed information about these 13 peptides is shown in **Table 1**.

**Table 1:** List of differential expressed peptides identified among all comparison pairs.

Precursor	Peptide sequence	AI *vs.* Con		CI *vs.* Con		AI *vs.* CI	
		Ratio	*P* value	Ratio	*P* value	Ratio	*P* value
Tubulin beta 1, 2A, 2B, 4B, 5 or 6	AVNMVPFPRLHFF	0.14	6.96E−05	0.22	8.81E−05	0.65	4.37E−02
Hemoglobin subunit beta-1 or 2	GHHLGKDFTPAAQAAFQKVVAGVATALAHKYH	0.17	1.36E−03	0.40	4.53E−03	0.43	1.34E−02
Glyceraldehyde-3-phosphate dehydrogenase	VKVGVNGFGRIGRLV	0.24	9.00E−05	0.093	6.00E−05	2.52	2.42E−02
60S ribosomal protein L13a	AEGQVLVLDGRGHLLG	0.35	1.15E−02	0.21	5.33E−03	1.67	1.39E−02
Prohibitin-2	AQNLKDLAGRLPAGPRGMGTAL	0.35	1.89E−03	0.22	7.52E−04	1.60	1.14E−02
Voltage-dependent anion-selective channel protein 2	AGGHKLGLALELEA	0.41	6.60E−03	0.27	2.41E−03	1.52	4.33E−02
Myelin basic protein	ILDSIGRFFSGDRGAPKRGSG	0.43	1.13E−02	0.27	4.48E−03	1.75	1.67E−02
Voltage-dependent anion-selective channel protein 3	AGGHKVGLGFELEA	0.49	8.45E−03	0.27	2.75E−03	1.86	4.55E−02
V-type proton ATPase subunit B, brain isoform	ALRAMRGIVNG	0.66	1.83E−02	0.35	2.84E−05	1.87	3.18E−02
Hemoglobin subunit alpha	ASLDKFLASVSTVLTSKYR	1.69	4.84E−02	3.36	1.10E−02	0.50	3.98E−02
Cytochrome c oxidase subunit 6A1, mitochondrial	SSGAHGEEGSARMWKALTYFVA	6.99	1.90E−04	3.67	2.86E−03	1.91	4.34E−03
WAS/WASL-interacting protein family member 3	PVPPPPPPPPPPPPPPPPPLG	8.26	4.84E−03	4.11	9.38E−03	2.01	4.41E−02
Phosphoglycerate mutase 1	FLGDEETVRKAMEAVAAQGKVK	7.88	2.56E−02	5.56	3.55E−05	1.87	4.66E−02

### Biological Function Analysis of Precursor Proteins of DEPs

**Figure 6:**
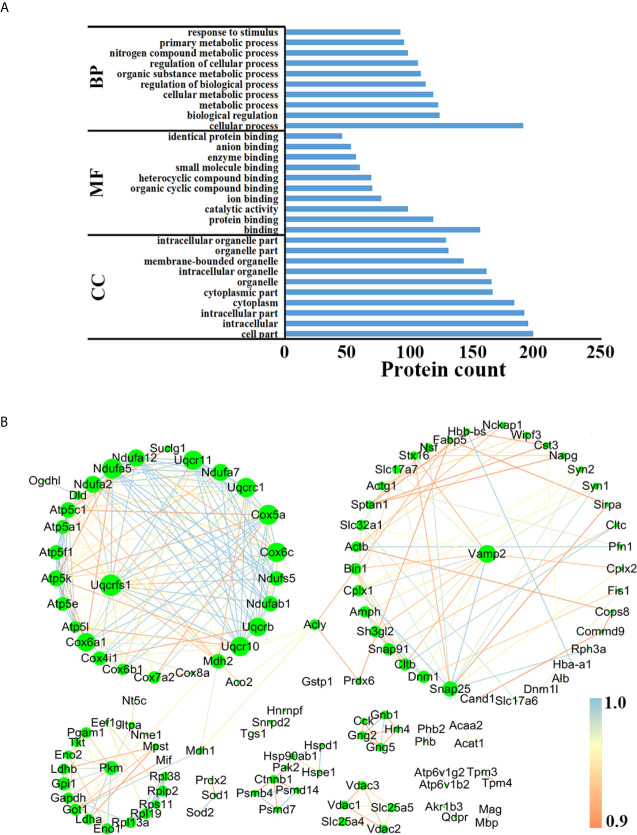
Functional analysis of the precursor protein of DEPs in the comparison between the AI and the control group. **(A)** GO analysis. **(B)** PPI analysis.

In order to better understand the biological functions of these DEPs, the precursor proteins of DEPs were analyzed using bioinformatic tools. As shown in **Figure 6A**, “cellular process”, “cell part” and “binding” in each module were the most highly enriched GO terms in the comparison between AI and control groups, while in the comparison between CI and control groups, the above three were also the most enriched GO terms (**Figure S3A**). Online STRING 11.0 was used to analyze the protein-protein interaction (PPI) among precursor proteins. As shown in **Figure 6B**, the PPI networks built based on DEPs identified between acutely infected mice and control mice include three larger clusters, and Rieske iron–sulfurprotein (UQCRFS1), vesicle-associated membrane protein 2 (Vamp2) and pyruvate kinase Pkm had the most connections in each cluster. Interestingly, Uqcrfs1 also had the most connections in the comparison between the CI and control group, as shown in **Figure S3B**.

### Novel Neuropeptide Identification

**Figure 7:**
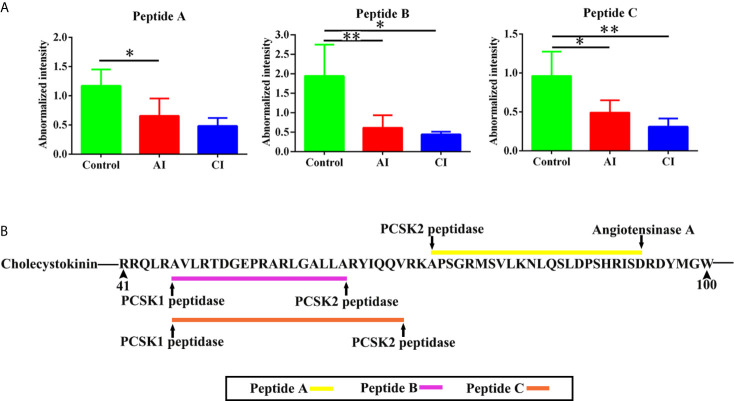
Identification of novel differentially expressed neuropeptides. **(A)** Normalized intensity of novel neuropeptides in different groups. **(B)** The prediction of possible proteases. Two-way ANOVA was used to determine statistical significance. *P* values of ≤0.05 (*), ≤0.01 (**), and ≤0.001 (***) were considered statistically significant.

Neuropeptides are important biological molecular that play key roles in cell–cell communication. As shown in **Figure 7A**, three novel neuropeptides were identified. These neuropeptides were derived from cholecystokinin. After *T. gondii* infection, peptide A [APSGRMSVLKNLQSLDPSHRISD], peptide B [AVLRTDGEPRARLGALLA] and peptide C [AVLRTDGEPRARLGALLARYIQQV] showed decreased expression levels when compared with the control group. As shown in **Figure 7B**, all possible proteases that might be involved in the induction of the above three neuropeptides were listed.

## Discussion

Previous studies showed that some neurobehavioral abnormities are associated with *T. gondii* infection (9–11). Multi-omics studies comprising transcriptomics and proteomics demonstrated *T. gondii*-induced metabolic reprogramming of human neuronal stem and monocytic cells. Besides, parasite phenotypic diversity plays a role in the reconstruction of the temporal signaling network in *Toxoplasma*-infected human brain cells (31). Peptidomics is the study of naturally endogenous peptides in biological samples (32). Identification of novel peptide hormones or neurotransmitters is important since those varied soluble molecules can give a wealth of knowledge concerning physical state, behavior modification and illness severity. Previously we developed a platform based on mass spectrometry for profiling endogenous serum peptides during toxoplasmosis (24). Our profiling study identified a large number of biologically active peptides that might be potential targets applicable to the diagnostics of toxoplasmosis. To investigate whether the brain peptidome was affected during toxoplasmosis development, we used the same sequencing strategy to perform a peptidomics study on mouse brain following *T. gondii* infection.

In the present study, we identified a total of 2,735 endogenous peptides which were derived from 612 precursor proteins. Our identification rate is about five times higher than a previously reported study conducted on mouse brains (33). A previous proteomic analysis identified 3,062 proteins in synaptosomes from *Toxoplasma*-infected and mock mice, of which 204 were precursor proteins found in the present study, which indicated a large number of endogenous peptides detected in this study were released from synaptosomes that synthesize, store, and release neurotransmitters (34). In the mouse serum, there were 607 soluble native peptides detected following *T. gondii* infection (24). However, only 62 peptides were overlapped in the two studies, which indicated that peptidome patterns were distinct in different tissues with *T. gondii* infection. Characterizations of the identified peptides revealed that the majority of their masses ranged from 1,000 to 3,500 Da, while their sizes ranged from 9 to 27 residues. Moreover, the frequency of the cleavage sites at N- or C-terminal varied in different physical stages. The endogenous peptide fragments are produced *via* peptide/protein processing *in vivo*, which are done by intracellular proteolysis or by extracellular protein decay by exocytosis (35). The endogenous peptides identified in this study therefore would provide potential candidates for bio-active neuropeptides.

With the progression of toxoplasmosis, a set of endogenous peptides in the infected brains were altered in their levels compared to the control samples, and the majority of the DEPs showed decreased levels. In the comparison between AI and control groups, expression levels of [KNIVTPRTPPPSQGKG], [RGSGKDSHTRTTHYGSLP] and [ASQKRPSQRSKYLATA] increased most dramatically. [RGSGKDSHTRTTHYGSLP], [SKYLATASTMDHAR] and [KNIVTPRTPPPSQGKG] were the top three with the most increased levels between CI and control groups. Interestingly, the above six peptides were derived from the same mother protein myelin basic protein (MBP). MBP with high abundance in CNS has been deemed as a marker of neuronal injury, as abnormality in its expression leads to hypomyelination and shivering symptoms (36). Although functional roles of MBP derived peptides are unknown, they are likely involved in physiological and behavioral processes during toxoplasmosis. Although there were 241 peptides identified in both acute and chronic phases, there were larger differences in peptide profiles between AI and control group than between CI and control group, which indicated that peptide profiling closely correlated with the pathophysiological features in brains affected by *T. gondii* infection. Unlike in the serum, at both acute and chronic infection stages DEPs in the brain mainly originated from cell part and took part in cellular process. Protein–protein interaction analysis of DEPs identified between acutely infected mice and control mice through their precursor proteins revealed three hub proteins, including Uqcrfs1, Vamp2 and Pkm. Uqcrfs1 was also the hub protein in the comparison between the CI and control group. *Uqcrfs1* conditional knockout mice showed lesions in the piriform area, which might result in lower nocturnal ambulatory movement and decreased performance in a motor coordination test in the rotarod (37). Vamp2 plays important roles in vesicular exocytosis and activity-dependent neurotransmitter release (38), which might suggest it is involved in neurological regulation during toxoplasmosis. PKM is glycolytic enzyme that catalyzes the transfer of a phosphoryl group from phosphoenolpyruvate to ADP, generating ATP. As the substrate of Parkin, PKM plays roles in early onset Parkinson’s disease and showed 2.2-fold higher levels in AD patients (39, 40). However, biological roles of Vamp2 along with Uqcrfs1 and Pkm in cerebral toxoplasmosis still need further exploration.

It is noteworthy that three novel neuropeptides were identified in this study. Peptide A [APSGRMSVLKNLQSLDPSHRISD], peptide B [AVLRTDGEPRARLGALLA] and peptide C [AVLRTDGEPRARLGALLARYIQQV] were derived from Cholecystokinin (CCK). CCK is involved in digestion and appetite *via* induction of gall bladder contraction and the release of pancreatic enzymes in the gut (41). There is also evidence to suggest that cholecystokinin may play a role in anxiety and panic disorders (42, 43). Following sequential proteolytic cleavages, CCK is cleaved posttranslationally to generate shorter peptides, such as CCK-8 and CCK-33 (44). Both CCK-8 and CCK-33 are gut–brain peptides that reduced food intake (45). Additionally, CCK-8 has a neuroprotective role as pretreatment of CCK-8 inhibited methamphetamine exposure induced behavioral and histologic changes (46). A number of animal tests have shown that mice infected with *T. gondii* showed significant decreased food intake and body weight loss. Besides, evidence is mounting that *Toxoplasma* multiplication and its persistence into brain have been linked to many behavioral and neurological disorders. Therefore, we speculate peptides A–C might be involved in host appetite, mental states and behavior. There are some shortages that limit the present study, which should be addressed in future work. First, optimal antibodies against peptide identified in this study should be produced, which will help determine the spatial and temporal expression pattern of interesting peptides in the brain in greater detail. Second, precursor proteins were significantly enriched in neurological diseases. However, further investigations are required to elucidate biological functions of these identified DEPs.

In conclusion, direct peptide profiling by mass spectrometry offers a means to further elucidate soluble molecular, especially neuropeptide regulation underlying brain functions. In this study, we firstly demonstrate herein alterations in peptidome of mouse brains infected with *T. gondii*, which will contribute to our knowledge of natural peptide content in the CNS. Data obtained in this study will advance our understanding of the roles of native peptides and their links in the behavior modifications during toxoplasmosis.

## Data Availability Statement

The mass spectrometry proteomics data have been deposited to the iProx (47) with the data set identifier PXD024506.

## Ethics Statement

The animal study was reviewed and approved by the Animal Ethics Committee of Lanzhou Veterinary Research Institute, Chinese Academy of Agricultural Sciences.

## Author Contributions

C-XZ, X-QZ, and H-YZ conceived and designed the experiments. C-XZ and MG performed the experiments. C-XZ, HC, and BH contributed reagents/materials/analysis tools. C-XZ and MG analyzed the data and wrote the paper. H-YZ and X-QZ critically revised the manuscript. All authors contributed to the article and approved the submitted version.

## Funding

Project support was provided by the National Natural Science Foundation of China (Grant Nos. 81971960 and 82002161), China Postdoctoral Science Foundation (Grant No. 2018M642663), the Agricultural Science and Technology Innovation Program (ASTIP) (Grant No. CAAS-ASTIP-2016-LVRI-03) and the QILU Young Scholars Program (Grant No. 21510082063092).

## Conflict of Interest

The authors declare that the research was conducted in the absence of any commercial or financial relationships that could be construed as a potential conflict of interest.

## Supplementary Material

Supplementary Figure 1 Statistics of peptides identified from brain and serum.

Supplementary Figure 2 Peptide profiles analysis between acutely infected group and chronically infected group. **(A)** Heat map of endogenous peptides. Columns were hierarchically clustered based on a complete linkage using Pearson correlation coefficients as the distance measure. **(B)** The volcano plot shows the individual statistically significant peptide between acutely infected group and chronically infected group. In this plot, the x-axis is log2 fold-change, which shows the direction of the change (negative scale is decrease and positive scale is increase) in the levels of peptide intensity, while the y-axis is the -log10 *p*-value, which shows the significance of the change.

Supplementary Figure 3 Functional analysis of the precursor protein of DEPs in the comparison between the CI and the control group. **(A)** GO analysis. **(B)** PPI analysis.

Supplementary Table 1 A list of peptides detected in mouse brain and serum.
