# Supplementary figures and images for: Quantitative Peptidomics of Mouse Brain After Infection With Cyst-Forming Toxoplasma gondii
Source: Front Immunol. 2021 Jul 22;12:681242. doi: 10.3389/fimmu.2021.681242 (PMC8340781; doi:10.3389/fimmu.2021.681242)

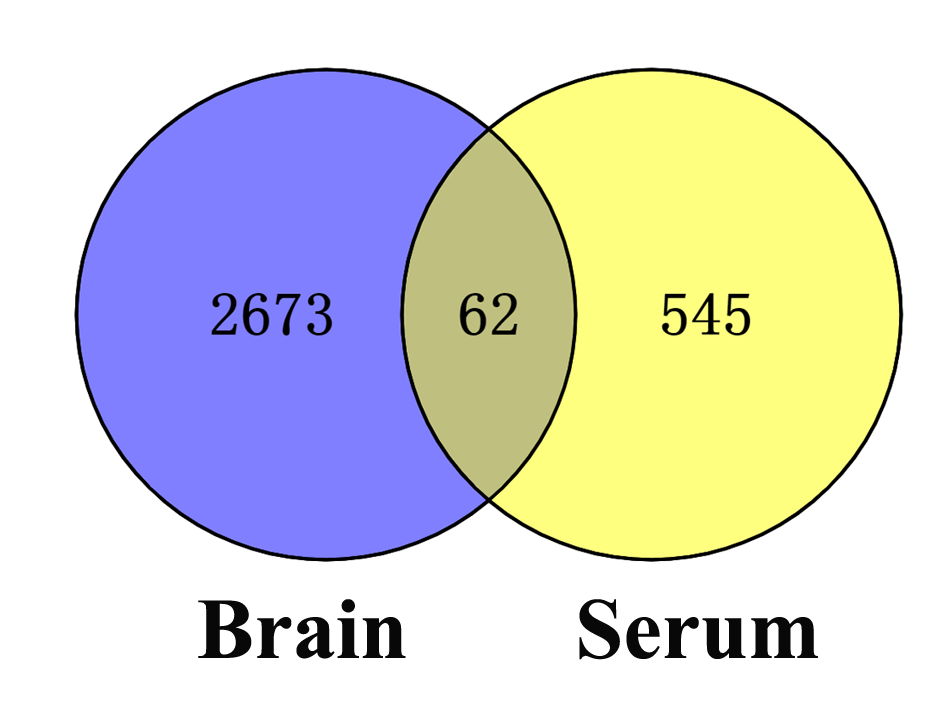

Supplement: Supplementary Figure 1 — Statistics of peptides identified from brain and serum. [file Image_1.tif]

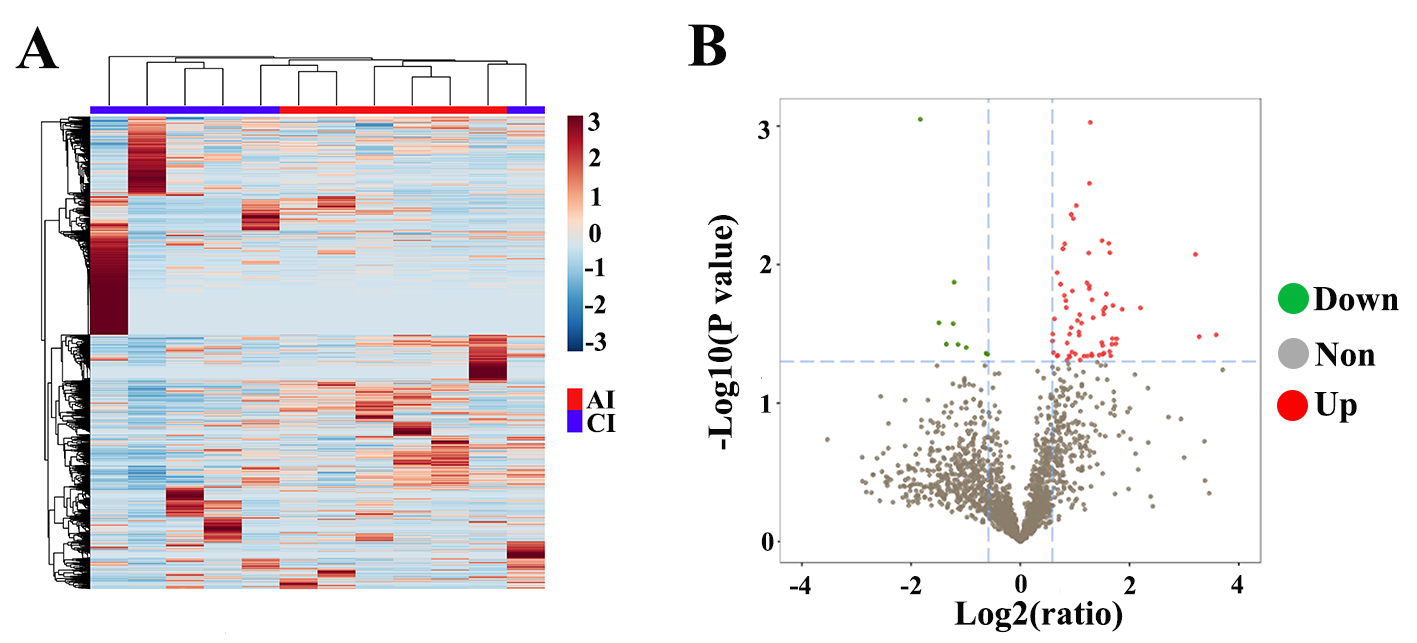

Supplement: Supplementary Figure 2 — Peptide profiles analysis between acutely infected group and chronically infected group. (A) Heat map of endogenous peptides. Columns were hierarchically clustered based on a complete linkage using Pearson correlation coefficients as the distance measure. (B) The volcano plot shows the individual statistically significant peptide between acutely infected group and chronically infected group. In this plot, the x-axis is log2 fold-change, which shows the direction of the change (negative scale is decrease and positive scale is increase) in the levels of peptide intensity, while the y-axis is the -log10 p-value, which shows the significance of the change. [file Image_2.tif]

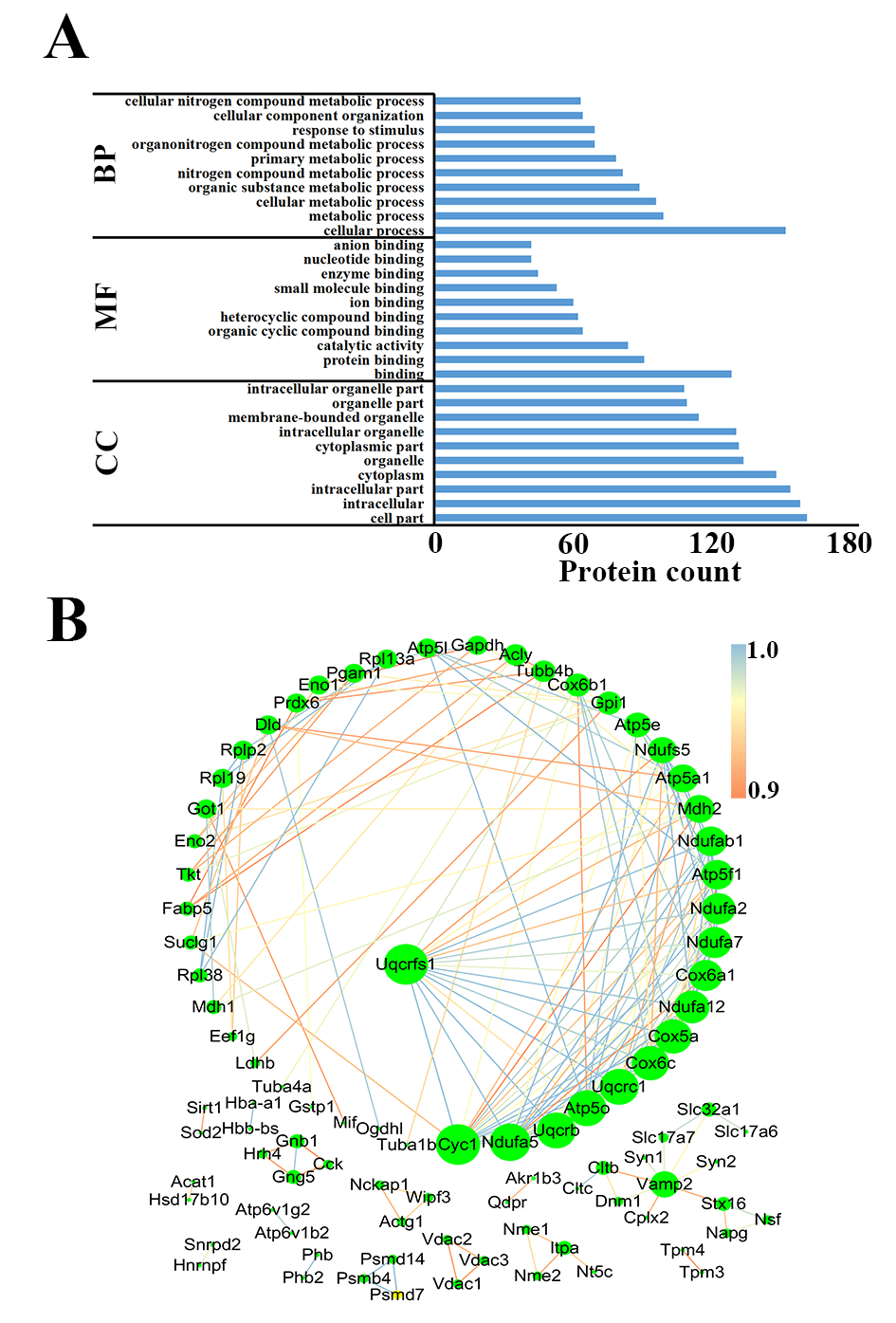

Supplement: Supplementary Figure 3 — Functional analysis of the precursor protein of DEPs in the comparison between the CI and the control group. (A) GO analysis. (B) PPI analysis. [file Image_3.tif]
